# Goal Management Training and Computerized Cognitive Training in Depression—a 2-Year Follow-Up of a Randomized Controlled Trial

**DOI:** 10.3389/fpsyt.2021.737518

**Published:** 2021-09-24

**Authors:** Bjørn Ingulfsvann Hagen, Jan Stubberud

**Affiliations:** ^1^Department of Research, Lovisenberg Diaconal Hospital, Oslo, Norway; ^2^Department of Psychology, University of Oslo, Oslo, Norway

**Keywords:** cognitive remediation, depression, long-term, follow-up, executive function

## Abstract

**Objective:** Information on the long-term effects of cognitive remediation (CR) in major depressive disorder (MDD) is lacking. The present study reports 2-year follow-up data from a previously published randomized controlled trial (RCT) from our research group, comparing Goal Management Training (GMT), a strategy-based CR intervention, to drill-and-practice computerized cognitive training (CCT). In previous work, we found comparable improvements in executive function (EF), in addition to reductions in depressive symptoms, following both GMT and CCT at 6-month follow-up.

**Methods:** Forty-two participants of the RCT, all diagnosed with MDD, were invited to complete rating-scales pertaining daily-life EF, rumination, and depressive symptoms. Explorative analyses compared the 2-year follow-up with previously published baseline and 6-month follow-up data, using non-parametric statistics. Similarly, GMT and CCT were compared at the 2-year follow-up, and completers were compared with non-completers.

**Results:** Twenty participants completed the study. Overall, completers (*n* = 20) and non-completers (*n* = 22) were similar. There were no significant differences between GMT (*n* = 11) and CCT (*n* = 9) for any outcome 2 years post-treatment. Reduction compared to baseline in depressive symptoms and rumination, but not in daily-life EFs, emerged for GMT only.

**Conclusions:** Findings suggest long-term improvements in mental health following GMT, while improvements in everyday EFs might require additional treatment or maintenance to sustain. Caution is warranted in the interpretation due to the small sample size and high attrition rates.

## Introduction

Major depressive disorder (MDD) is a prevalent and debilitating condition that often includes a cyclic course of remission and relapse (1). Deficits in cognitive functions, including executive functions (EFs), are common in MDD, and frequently remain following the alleviation of affective symptoms (2, 3). These deficits, particularly executive dysfunction, negatively impact daily-life functioning and are associated with impaired emotion regulation capacities and increased levels of rumination (4, 5). Accordingly, impairments in EFs represent a risk factor for subsequent relapse and are central to long-term outcomes in MDD (6).

Cognitive remediation (CR) refer to a set of interventions aiming to produce durable and generalizable improvements in cognition, and in broad terms comprise two approaches, that are often combined. In short, drill-and-practice approaches involve exercises to improve cognitive functions, while strategy-based CR aim to promote the application of compensatory strategies in daily life (7). Overall, CR have yielded promising short-term results in MDD (8, 9). A recent meta-analysis including both drill-and-practice- and strategy-based approaches found a moderate effect of CR on cognitive functioning, including EFs, and small effects on depressive symptoms and daily functioning, when compared to non-CR control conditions (7). However, the improvements did not remain at follow-up, even if reassessments have typically been conducted 1–3 months post-treatment. The lack of durable treatment effects could partly be due to the limited number of studies with additional follow-up assessments (7). Furthermore, comparisons of long-term outcomes following drill-and-practice- and strategy-based approaches are lacking.

To our knowledge, only a couple of studies have evaluated the effectiveness of CR beyond 3 months. In a 1-year follow-up of a randomized controlled trial (RCT), Hoorelbeke et al. (10) identified reductions in depressive symptoms and rumination, and improved perceived daily-life EF, in a remitted depressed sample following drill-and-practice CR targeting executive processes. Still, no differences between the groups, a high cognitive load intervention group vs. a low cognitive load control group, emerged. Similarly, in a mixed sample of 153 participants (schizophrenia/schizoaffective disorder, bipolar disorder, depression), including MDD (*n* = 58), Twamley et al. (11) found improvements in cognitive functioning, depressive symptoms, and multiple measures of daily functioning, 2 years following CR. Interestingly, results were similar for strategy-based CR combined with supported employment and an enhanced supported employment control group (11).

The present study report 2-year follow-up data from a RCT (*n* = 63) comparing a strategy-based CR intervention for improving EFs, Goal Management Training–GMT (12), with drill-and-practice computerized cognitive training (CCT), in a depression sample. In the RCT performed by our research group, improvements in performance-based measures of EFs and reductions in rumination were observed for both groups, with effects lasting at least 6 months post-treatment (13). At the same time, only participants in the GMT-group reported significant improvements in perceived daily-life EFs and reductions in depressive symptoms between baseline and the 6-month follow-up, even though between-group differences remained non-significant. Overall, these findings align with a recent meta-analysis including various neurocognitive disorders concluding that GMT is moderately effective in improving EFs and aspects of mental health (14). Nevertheless, only one study has reported follow-up analyses more than 2 years following GMT (15). Here, our research group found that improvements following GMT in EF and quality of life at 6-months follow-up, were no longer present at 5-years follow-up (15–17). In a similar vein, the lack of long-term follow-up studies is a limitation in the current literature on CR in MDD and likely represents an obstacle for clinical implementation (7, 9). Hence, the aim of the present study was to evaluate the long-term effects of CR, and concurrently compare a strategy-based approach to a drill-and-practice approach, in this context.

## Materials and Methods

### Procedures

Participants of the original RCT (clinical.trials.gov identifier NCT03338413) had all received a diagnosis of mild or moderate MDD according to ICD-10 criteria, either as primary- or secondary diagnosis, by a clinical psychologist during outpatient treatment that predated study participation by 0–24 months. No diagnostic reassessment was done prior to participation in either the original study, or at the 2-year follow-up. Additional inclusion criteria were self-reported EF difficulties on a customized telephone interview and being 18–60 years of age. Exclusion criteria included neurological conditions, drug use, lack of proficiency in Norwegian, or severe cognitive problems or mental disorders.

Those who completed the CR and the 6-month follow-up assessment of the original RCT (*n* = 42) were invited to participate in the present 2-year follow-up study through letters (two letters—an invitation, and a reminder 2 weeks later). Participation involved completing an online questionnaire comprising rating-scales addressing perceived daily-life EF, rumination, and depressive symptoms. The data were collected (November 2020–January 2021) and stored using the Service for Sensitive Data facilities, owned by the University of Oslo. All participants provided written informed consent, electronically signed, and collected using the same platform. A clinical psychologist was available for questions or concerns during participation. The project, including the 2-year follow-up, was approved by the Regional Committee for Medical and Health Research Ethics, South-Eastern Norway (identifier 2017/666).

### Interventions

GMT is a manual-based metacognitive CR intervention aiming to improve EFs through teaching compensatory strategies, particularly directed at problem-solving and inhibition capacities (12). Mindfulness techniques for promoting sustained and focused attention are also included (14). In the present study, the training additionally comprised elements of cognitive restructuring of negative thoughts. The CCT consisted of seven exercises from the BrainHQ platform, addressing processing speed, social cognition, and EFs (attention and working memory). Both treatments entailed nine sessions, but the session frequency, treatment duration, group-sizes, and total hours of training differed (GMT = 2 h once a week for 9 weeks, 5–7 participants; CCT = 1 h twice a week for 4.5 weeks, 2–3 participants). See Hagen et al. (13) for a more detailed description of the study.

### Measures

Perceived daily-life EF was measured using the 75-item Behavior Rating Inventory of Executive Function—Adult Version–BRIEF-A (18). The BRIEF-A consist of three index scores, the Behavioral Regulation Index (BRI; 30 items, total range = 30–90) and the Metacognition Index (MI; 40 items, total range = 40–120), which are combined to make the Global Executive Composite (GEC; total range = 70–210). The Beck Depression Inventory–BDI (19) was used to measure depressive symptom severity, with respondents indicating on a four-point scale (item range = 0–3; total range: 0–63) the extent to which they have experienced depressive symptoms during the previous week. The total score from the 22-item Ruminative Response Scale–RRS (20) was used to assess rumination (item range = 1–4). The Color-Word Interference Test—condition 4 from the Delis-Kaplan Executive Function System–D-KEFS (21), collected as part of the original RCT, was included as a performance-based measure of EF when comparing completers and non-completers.

### Statistical Analyses

Completers and non-completers in the 2-year follow-up were compared, using the non-parametric Mann-Whitney U test, on previously collected data from baseline and the 6-month follow-up using demographic variables, a performance-based measure of EF, and the included outcome variables, in addition to change scores between baseline and the 6-month follow-up. Similarly, GMT and CCT were compared at the 2-year follow-up. For the whole sample, GMT, and CCT, 2-year follow-up data was compared with data from baseline and the 6-month post-treatment using the Wilcoxon signed-rank test.

An additional sensitivity analysis to ascertain the robustness of the conclusion, including all invited participants, was conducted for the BRIEF-A GEC, BDI, and RRS, using a linear mixed model for repeated measures. The model had an unstructured covariance matrix to estimate both within- and between effects. Group, Time, and Group-Time interactions were included as fixed group differences, and the restricted maximum likelihood method was used for estimation. The significance-level was *p* < 0.05 for all tests.

## Results

Twenty participants (GMT, *n* = 11; CCT, *n* = 9) completed the 2-year follow-up, totalling 47.6% of the invited participants. Completers did not differ significantly from non-completers for most outcomes, neither at baseline, nor for change scores between baseline and the 6-month follow-up. One exception was that non-completers reported significantly higher levels of rumination at baseline (Table 1).

**Table 1:** Characteristics of completers and non-completers of the 2-year follow-up.

**Variable**	**Completers (*n =* 20)**	**Non-completers (*n =* 22)**	***p***
	**Mean (*SD)***	**Mean (*SD)***	
**Baseline**			
Age	43.2 (9.1)	41.8 (8.5)	0.724
Estimated IQ	114.1 (6.8)	109.0 (13.2)	0.434
BRIEF-A GEC	132.3 (18.8)	132.0 (14.9)	0.791
BDI	16.9 (6.9)	16.6 (7.3)	0.850
RRS	51.5 (10.7)	59.3 (10.8)	0.049^*^
CWIT 4 (sec)	56.9 (9.5)	59.2 (11.1)	0.743
**Change scores**			
BRIEF-A GEC change	13.2 (22.0)	9.0 (16.0)	0.427
BDI change	6.5 (6.6)	2.4 (6.8)	0.112
RRS change	9.7 (10.7)	11.9 (11.1)	0.687
CWIT 4 change (sec)	7.7 (7.2)	9.2 (7.8)	0.553

* *p < 0.05. Change scores are calculated by subtracting 6-month follow-up scores from the baseline scores. The two-subscale version of the Wechsler Abbreviated Scale of Intelligence was used to estimate IQ. All scores are raw scores, except IQ. BRIEF-A GEC, Behavior Rating Inventory of Executive Function—Adult version Global Executive Composite; BDI, Beck Depression Inventory; RRS, Ruminative Response Scale; CWIT 4, Color-Word Interference Test—Condition four; Sec, seconds*.

Compared to baseline, whole sample analysis (*n* = 20) revealed significant improvements in depressive symptoms and rumination, but not in perceived daily-life EF at the 2-year follow-up (Table 2). Similar results emerged for GMT, while no statistically significant changes emerged following CCT. No significant changes emerged for any group between the 6-month follow-up and the 2-year follow-up. Lastly, no significant between-group differences (GMT/CCT) emerged for any outcome at the 2-year follow-up (Table 2), and the sensitivity analysis produced similar results (Table 3). The data from baseline and the 6-month follow-up reported in Tables 1, 3 include subsets of data previously published in Hagen et al. (13).

**Table 2:** Two-year follow-up outcomes for GMT, CCT, and the whole sample.

**Variable**	**GMT (*n =* 11)**	**CCT (*n =* 9)**	***p***	**Total (*n =* 20)**
	**Mean (*SD)***	**Mean (*SD)***		**Mean (*SD)***
BRIEF-A GEC	122.0 (22.9)	128.9 (24.9)	0.382	125.1 (23.4)
BRIEF-A MI	73.6 (16.2)	76.4 (19.1)	0.470	74.9 (17.2)
BRIEF-A BRI	48.5 (8.7)	52.4 (8.6)	0.341	50.3 (8.7)
BDI	8.8 (6.9)^**^	14.8 (9.4)	0.148	11.5 (8.5)^*^
RRS	38.6 (7.1)^**^	42.4 (12.1)	0.196	40.4 (9.6)^**^

*Significant within-group change compared with baseline*,

* *p < 0.05*,

** *p < 0.01. GMT, Goal Management Training; CCT, Computerized cognitive training; BRIEF-A, Behavior Rating Inventory of Executive Function – Adult version; GEC, Global Executive Composite; MI, Metacognitive Index; BRI, Behavioral Regulation Index; BDI, Beck Depression Inventory; RRS, Ruminative Response Scale*.

**Table 3:** Fixed effect estimates from the intention-to-treat linear mixed model sensitivity analysis.

		**Baseline**	**Six-month follow-up**	**Two-year follow-up**	**Group**	**Time**	**Group-time**
**Measure *M* [95 % CI]**		**T1**	**T2**	**T3**	***p***	***p***	***p***
BRIEF-A GEC	GMT	134.1 [127.3, 140.1]	119.3 [110.3, 128.4]	122.8 [109.8, 135.8]	0.751	0.004	0.232
	CCT	129.5 [121.9, 137.2]	123.3 [113.3, 133.3]	128.7 [114.3, 143.1]			
BDI	GMT	17.2 [14.3, 20.1]	12.3 [8.7, 15.9]	8.8 [3.9, 13.6]	0.322	0.001	0.055
	CCT	16.1 [12.8, 19.4]	12.4 [8.4, 16.3]	16.4 [11.0, 21.7]			
RRS	GMT	56.0 [51.2, 60.7]	44.8 [40.1, 49.6]	40.5 [35.0, 45.9]	0.610	<0.001	0.240
	CCT	55.3 [49.9, 60.6]	44.7 [39.4, 50.0]	45.9 [39.9, 52.0]			

*GMT, Goal Management Training; CCT, Computerized cognitive training; BRIEF-A, Behavior Rating Inventory of Executive Function – Adult version; GEC, Global Executive Composite; BDI, Beck Depression Inventory; RRS, Ruminative Response Scale*.

## Discussion

The aim of the present study was to evaluate the long-term effectiveness of CR in improving daily-life EF and reducing depressive symptoms and rumination, in MDD. Importantly, the number of non-completers exceeded the cut-offs for when attrition represents a threat to validity, increasing the risk of bias and challenging generalizability (22). Taken together with the small sample size, all statistical analyses should be considered exploratory, and findings are to be interpreted with caution due to the high attrition rates and low statistical power.

In contrast to the recent meta-analysis by Legemaat et al. (7), the present study identified long-term improvements in depressive symptoms, in addition to reduced rumination, following CR. Further, results suggest that these improvements are specific to GMT, and as such provide preliminary support of strategy-based CR being more effective in producing long-term improvements in mental health. Changes in perceived everyday EF did not sustain over the course of years in the present study, like previous evaluations of long-term effects following GMT (15). Concurrently, our findings align with previous research failing to find evidence of different long-term effectiveness between treatments (10, 11). On the other hand, the results are in contrast with Hoorelbeke et al. (10), where durable reductions in daily-life EF difficulties, depressive symptoms, and rumination following drill-and-practice CCT were reported.

The present study highlights the challenges associated with conducting longitudinal clinical studies. There is a need for high-quality reports on long-term effects beyond 6-months, as CR interventions in MDD is yet to provide convincing evidence of durable effects (7). A first step toward this goal is to plan and conduct large-scale studies with adequate statistical power (9, 22). Indeed, the sample sizes in previous research, particularly for interventions requiring considerable therapist involvement, have limited the prospects of conducting long-term follow-ups (7, 8). Steps for optimizing retention could additionally prove useful for attaining valid results over the course of years (23).

Findings from the present study does not indicate that patient characteristics reliably predict retention in CR. Previous research has identified disappointment with the perceived profits from treatment as one of the most common side-effects in CR (24), which may influence the commitment to complete follow-up assessments. Still, our exploratory analyses did not indicate that this was the case as neither self-reported nor performance-based improvement following treatment differed between completers and non-completers.

### Limitations

In addition to the concerns related to the sample size, attrition rates, and lack of diagnostic reassessment, the following limitations should be noted. No performance-based measures were applied at the 2-year follow-up, and the study relied on self-report. Importantly, the outcome of the RCT was available to the participants, potentially introducing bias through revealing the active treatment. The GMT and CCT differed in treatment duration and frequency of sessions, challenging the comparison between the two, and the study lacked a no-intervention control group to isolate the effect of *Time*.

## Conclusions

Findings suggest that improvements in mental health remain long-term following GMT, while enhanced daily-life EF might require additional treatment or maintenance (e.g., booster-sessions) to sustain. At the same time, results highlight a potential for increasing the durability of CR treatment effects. Future research should take measures to ensure high-quality results concerning long-term outcomes.

## Data Availability Statement

The dataset for this article is not publicly available because of restrictions specified in the study consent-form, and conditions for approval from the local ethics committee, concerning patient confidentiality and participant privacy. Requests to access the data that support the findings of this study should be directed to author JS (j.e.stubberud@psykologi.uio.no).

## Ethics Statement

The studies involving human participants were reviewed and approved by Regional Committee for Medical and Health Research Ethics, South-Eastern Norway (identifier 2017/666). The patients/participants provided their written informed consent to participate in this study.

## Author Contributions

JS and BH contributed to the conceptualization of the study. JS contributed to the funding acquisition and acted as study PI. BH completed data collection, was responsible for data curation, and wrote the original article draft. Both authors contributed with revision of the original article draft and have approved the final manuscript.

## Funding

This study was funded by The South-Eastern Norway Regional Health Authority (Grant No. 2019120) and the research fund at Lovisenberg Diaconal Hospital.

## Conflict of Interest

The authors declare that the research was conducted in the absence of any commercial or financial relationships that could be construed as a potential conflict of interest.

## Publisher's Note

All claims expressed in this article are solely those of the authors and do not necessarily represent those of their affiliated organizations, or those of the publisher, the editors and the reviewers. Any product that may be evaluated in this article, or claim that may be made by its manufacturer, is not guaranteed or endorsed by the publisher.
